# Development and Validation of a Novel Food-Based Global Diet Quality Score (GDQS)

**DOI:** 10.1093/jn/nxab244

**Published:** 2021-10-23

**Authors:** Sabri Bromage, Carolina Batis, Shilpa N Bhupathiraju, Wafaie W Fawzi, Teresa T Fung, Yanping Li, Megan Deitchler, Erick Angulo, Nick Birk, Analí Castellanos-Gutiérrez, Yuna He, Yuehui Fang, Mika Matsuzaki, Yiwen Zhang, Mourad Moursi, Selma Kronsteiner-Gicevic, Michelle D Holmes, Sheila Isanaka, Sanjay Kinra, Sonia E Sachs, Meir J Stampfer, Dalia Stern, Walter C Willett

**Affiliations:** Harvard T.H. Chan School of Public Health, Boston, MA, USA; National Institute of Public Health, Cuernavaca, Mexico; Harvard T.H. Chan School of Public Health, Boston, MA, USA; Channing Division of Network Medicine, Brigham and Women's Hospital, Boston, MA, USA; Harvard T.H. Chan School of Public Health, Boston, MA, USA; Harvard T.H. Chan School of Public Health, Boston, MA, USA; Department of Nutrition, Simmons University, Boston, MA, USA; Harvard T.H. Chan School of Public Health, Boston, MA, USA; Intake - Center for Dietary Assessment, FHI Solutions, Washington, DC, USA; National Institute of Public Health, Cuernavaca, Mexico; Harvard T.H. Chan School of Public Health, Boston, MA, USA; National Institute of Public Health, Cuernavaca, Mexico; National Institute for Nutrition and Health, Chinese Center for Disease Control and Prevention, Beijing, China; National Institute for Nutrition and Health, Chinese Center for Disease Control and Prevention, Beijing, China; Johns Hopkins Bloomberg School of Public Health, Baltimore, MD, USA; Harvard T.H. Chan School of Public Health, Boston, MA, USA; Intake - Center for Dietary Assessment, FHI Solutions, Washington, DC, USA; Harvard T.H. Chan School of Public Health, Boston, MA, USA; London Centre for Integrative Research on Agriculture and Health, London, United Kingdom; Harvard T.H. Chan School of Public Health, Boston, MA, USA; Channing Division of Network Medicine, Brigham and Women's Hospital, Boston, MA, USA; Harvard T.H. Chan School of Public Health, Boston, MA, USA; London School of Hygiene and Tropical Disease, London, United Kingdom; Center for Sustainable Development, Columbia University, New York, NY, USA; Harvard T.H. Chan School of Public Health, Boston, MA, USA; Channing Division of Network Medicine, Brigham and Women's Hospital, Boston, MA, USA; National Institute of Public Health, Cuernavaca, Mexico; Harvard T.H. Chan School of Public Health, Boston, MA, USA; Channing Division of Network Medicine, Brigham and Women's Hospital, Boston, MA, USA

**Keywords:** diet quality metrics, dietary diversity, nutrient adequacy, noncommunicable disease, double burden of malnutrition, nutrition transition, nutritional epidemiology, monitoring and evaluation, nutrition surveillance, GDQS

## Abstract

**Background:**

Poor diet quality is a major driver of both classical malnutrition and noncommunicable disease (NCD) and was responsible for 22% of adult deaths in 2017. Most countries face dual burdens of undernutrition and NCDs, yet no simple global standard metric exists for monitoring diet quality in populations and population subgroups.

**Objectives:**

We aimed to develop an easy-to-use metric for nutrient adequacy and diet related NCD risk in diverse settings.

**Methods:**

Using cross-sectional and cohort data from nonpregnant, nonlactating women of reproductive age in 10 African countries as well as China, India, Mexico, and the United States, we undertook secondary analyses to develop novel metrics of diet quality and to evaluate associations between metrics and nutrient intakes and adequacy, anthropometry, biomarkers, type 2 diabetes, and iteratively modified metric design to improve performance and to compare novel metric performance to that of existing metrics.

**Results:**

We developed the Global Diet Quality Score (GDQS), a food-based metric incorporating a more comprehensive list of food groups than most existing diet metrics, and a simple means of scoring consumed amounts. In secondary analyses, the GDQS performed comparably with the Minimum Dietary Diversity - Women indicator in predicting an energy-adjusted aggregate measure of dietary protein, fiber, calcium, iron, zinc, vitamin A, folate, and vitamin B12 adequacy and with anthropometric and biochemical indicators of undernutrition (including underweight, anemia, and serum folate deficiency), and the GDQS also performed comparably or better than the Alternative Healthy Eating Index - 2010 in capturing NCD-related outcomes (including metabolic syndrome, change in weight and waist circumference, and incident type 2 diabetes).

**Conclusions:**

The simplicity of the GDQS and its ability to capture both nutrient adequacy and diet-related NCD risk render it a promising candidate for global monitoring platforms. Research is warranted to validate methods to operationalize GDQS assessment in population surveys, including a novel application–based 24-h recall system developed as part of this project.

## Introduction

Dietary factors are responsible for a greater fraction of global age-standardized adult mortality (22% of total deaths among those aged ≥25 y) than any other risk factor (1). Most diet-related mortality is caused by cardiovascular disease, type 2 diabetes, and cancer (1), and 82% of diet-related deaths now occur in low- and middle-income countries (LMIC) (2). In addition to dietary imbalances contributing to metabolic risks and noncommunicable disease (NCD) mortality, protein, energy, and micronutrient deficits continue to contribute to a dual burden of undernutrition in most LMIC and further threaten health and livelihoods, particularly those of women and children (3).

Valid, robust, and easily operationalized diet metrics are needed to systematically assess, compare, and track risks of nutrient inadequacy and diet-related NCDs in populations and to inform effective, evidence-based policies and programs for improving diet quality (4). Existing metrics have focused on specific dimensions of diet quality (such as nutrient adequacy or NCD risk) or specific (usually high-income) populations, or have high data needs (such as the use of food composition data for analysis) that are not conducive to applications in limited-resource settings (5). Conversely, existing metrics that are feasible to collect in limited-resource settings typically involve simplistic ways of scoring amounts of foods consumed, which limit metric performance (5). Without a standard, validated global metric that is feasible to collect in limited-resource settings and that can sensitively measure diet quality in terms of both nutritional adequacy and NCD risk, the international community and UN Sustainable Development Goals lack a critical component of global health surveillance.

In 2018, Intake *–* Center for Dietary Assessment launched a 2-y research initiative to help fill this gap. The aim of the initiative was to develop a novel suite of simple yet robust metrics for assessing diet quality at the population level in diverse LMIC. Such metrics were intended to be appropriate for within- and between-population comparisons and tracking over time, applicable to program monitoring, evaluation, design, communication, and advocacy, and inherently simple and inexpensive to collect and analyze, and to have consistent interpretation across settings and potential for integration in existing data collection platforms and surveillance systems. The metrics to be developed were to be appropriate for use among nonpregnant, nonlactating women of reproductive age, considering the importance of this group as a major focus of nutrition interventions globally (4), but ideally would also be applicable to other demographic groups and high-income countries as all nations are included in the Sustainable Development Goals.

Following a competitive solicitation process, Intake selected a team at Harvard University to lead the research initiative. The team worked collaboratively with researchers from the National Institute of Public Health, Mexico; the Chinese Center for Disease Control and Prevention; the London School of Hygiene and Tropical Medicine; the Center for Sustainable Development at Columbia University; the Addis Continental Institute of Public Health, Ethiopia; and Intake to carry out the requested metric development work. This paper provides a broad overview of the approach and results of metric development, whereas other papers in this Supplemental Issue describe detailed evaluations of metric performance in cross-sectional (6–10) and cohort (11–13) datasets.

## Methods

### Starting point for metric development: the Prime Diet Quality Score

We selected the Prime Diet Quality Score (PDQS) (15) as the initial basis for the development of novel metrics. The PDQS is a food-based metric of diet quality that includes 21 food groups, 14 of which are classified as healthy and 7 as unhealthy based on review of the literature on dietary contributors to nutrient intakes and NCD risk globally (16, 17). Healthy food groups are assigned more points for higher consumption (0 points for 0–1 servings/wk, 1 point for 2–3 servings/wk, and 2 points for 4+ servings/wk). Scoring is reversed for unhealthy groups (more points are given for lower consumption). The PDQS food-based design, differentiation of healthy and unhealthy foods groups, and modestly expanded list of food groups compared with most existing metrics (5) allow it to be applied to a range of global diets and capture the contribution of diet to both undernutrition and NCD risk without requiring food composition data in analysis. The metric's trichotomous approach to scoring consumed amounts also provides a potentially more sensitive, though not overly complicated, means of capturing diet quality than some existing metrics. Analyses have found higher PDQS scores to be correlated with key nutrient intakes in US women (18) and inversely associated with incident heart disease, gestational diabetes, hypertension, and all-cause mortality in US adults (15, 19, 20), cardiovascular risk factors among older Spanish adults (21, 22), and preterm birth, low birth weight, and fetal loss in pregnant Tanzanian women (23).

### Modifications to PDQS food groups

In developing candidate diet quality metrics to be tested, we first undertook modifications to the list of PDQS food groups to represent the diversity of nutritionally important foods more fully across LMIC globally, and the most up-to-date scientific evidence regarding relations between different foods and health. Major changes included the following:

- Removing “carrots” and adding 3 new deep orange food groups (deep orange fruits, vegetables, and tubers);
- Expanding “poultry” to also include lean game meats;
- Expanding “fish” to also include shellfish and other important seafood contributors to n-3 fatty acids and protein;
- Modifying “fried foods away from home” to specifically target deep fried foods that are purchased;
- Treating “eggs” [which we have sometimes not included as a scored component in adults (18, 20, 24)] as a healthy food group;
- Adding a positively scored “low fat dairy” group;
- Modifying the scoring approach for high fat dairy and red meat so that increasing points are given until specific consumed amounts, after which no points are given, to recognize modest consumption of these groups as an important source of nutrients and higher consumption as an NCD risk factor;
- Adding juice (defined as any unsweetened or sweetened drink at least partly composed of fruit juice) as an unhealthy group.

Rationales for the inclusion and scoring approach of the 25 food groups retained in the final metric, details on the operational definition of each food group, and rationales for excluding certain food groups are provided in **Supplemental Methods**.

### Modifications to the PDQS scoring method

We also modified the trichotomous basis upon which PDQS food groups are scored from *servings* per day to *grams* per day, to facilitate more comparable assessments across countries and over time. We selected the gram per day cutoffs for each food group based on their ability to produce a reasonably even distribution of categories of consumed amounts of each food group based on analysis of FFQ and 24-h recall (24HR) data from cross-sectional and cohort studies of nonpregnant and nonlactating women in diverse settings (**Table 1**). We implemented further minor adjustments to these cutoffs to facilitate primary data collection, following methodology described by Moursi and colleagues in this Supplemental Issue (14).

**TABLE 1:** Summary of datasets used to develop and evaluate metrics^1^

Diet methods and sample	Foods included in data, *n*	Reference period or no. of 24HRs	Portion size information	FFQ frequency options (if applicable)	Outcomes
Cross-sectional datasets					
Millennium Villages Project (10 Sub-Saharan African countries) (25)					
FFQ from 1624 rural NPNL WRA; separate instrument developed for each village	92–161, depending on country	Past month	Nonquantitative (no portion size information)	Never, 1/mo, 2–3/mo, 1/wk, 2–3/wk, 4–6/wk, 1/d, ≥2/d	Nutrient intake and adequacy, BMI, MUAC, hemoglobin
Anemia etiology in Ethiopia study (26)					
FFQ from 1604 mostly rural NPNL WRA^2^	454	Past week	Quantitative: 7 food item–specific portion sizes assessed for each food	Never, 1/wk, 2–4/wk, 5–6/wk, 1/d, 2–3/d, 4–5/d, ≥6/d	Nutrient intake and adequacy, BMI, MUAC, hemoglobin, ferritin, serum folate, serum vitamin B12, blood pressure
24HR from 1593 mostly rural NPNL WRA^2^	113	1 24HR, and 2nd in subset of participants	Multiple-pass probe incorporating information on no. of meals at which each food was consumed, no. of servings of each food consumed at each meal, and average portion size of each food	NA	Same as above
2010–2012 China National Nutrition and Health Survey (27)					
24HR from 15,173 urban and rural NPNL WRA	1615	3 consecutive d (2 wkd and 1 wkend)	Quantitative: estimated g consumed/last 24 h each d of the 3 d	NA	Nutrient intake and adequacy, BMI, waist circumference, hemoglobin, glucose, HDL and total cholesterol, triglycerides, blood pressure, metabolic syndrome
Indian Migration Study and Andhra Pradesh Children and Parents Study (28, 29)					
FFQ from 3065 mostly rural NP WRA ^3^	184	Past year	Portion size estimates with quantitative: standard household utensils (e.g., tablespoon, ladle, and bowl), data on no. of portion sizes consumed also collected	Never, yearly, monthly, weekly, daily	Nutrient intake and adequacy, BMI, hemoglobin, HDL and total cholesterol, blood pressure
2012 and 2016 Mexican National Surveys of Health and Nutrition (30, 31)					
FFQ from 4975 urban and rural NPNL WRA^2^	140	Past week	Quantitative: 2–3 portion sizes offered for each food, data on no. of portion sizes consumed also collected	Never, 1/wk, 2–4/wk, 5–6/wk, 1/d, 2–3/d, 4–5/d, ≥6/d	Nutrient intake and adequacy, BMI, waist circumference, hemoglobin, ferritin, serum folate, serum vitamin B12, glucose, insulin, LDL cholesterol, HDL cholesterol, total cholesterol, triglycerides, metabolic syndrome
24HR from 2545 urban and rural NPNL WRA^2^	544	1 24HR, 2nd in subset of participants	Multiple 5-pass probe incorporating weighed amounts or common household measurement implements	NA	Same as above
Cohort datasets					
Mexican Teachers Cohort (32)					
FFQ from 8967 urban and rural NPNL WRA	125	Past year	Semiquantitative: standard portion size or commonly used unit indicated	Never, ≤1/mo, 2–3/mo, 1/wk, 2–4/wk, 5–6/wk, 1/d, 2–3/d, 4–5/d, ≥6/d	Weight change, waist circumference change
US Nurses' Health Study II (33)					
FFQ from 56,321 urban and rural NP WRA^3^	135	Past year	Semiquantitative: standard portion size or commonly used unit indicated	Never or <1/mo, 1–3/mo, 1/wk, 2–4/wk, 5–6/wk, 1/d, 2–3/d, 4–5/d, ≥6/d	Weight change, incident type 2 diabetes

1 In cross-sectional datasets, sample size corresponds to the number of participants with dietary data (for some outcomes, available sample size was smaller; refer to (25–33) for more details. MUAC, mid–upper arm circumference; NA, not applicable; NP, nonpregnant; NL, nonlactating; WRA, women of reproductive age; 24HR, 24-hour recall.

2 FFQ and 24HR data from the Anemia Etiology in Ethiopia Study were collected from the same sample. FFQ and 24HR data from the 2012 and 2016 Mexican National Surveys of Health and Nutrition were collected from separate samples.

3 The Indian Migration Study and Andhra Pradesh Children and Parents Study population consists of NP WRA (lactation was not ascertained). In analysis of the Nurses’ Health Study II, women were classified as NP (lactation was not ascertained), but 2-y time periods during which a pregnancy was reported were excluded from analysis to limit the influence of lactation.

### Refinement of candidate metrics

Upon implementing initial refinements to PDQS food group definitions and scoring to create an updated PDQS-like Metric, we scored the metric using FFQ, 24HR, or both FFQ and 24HR measurements in each dataset (Table 1); evaluated associations between the PDQS-like Metric and outcomes available in each dataset using Spearman correlations and multivariable regression models to examine trends in metric–outcome associations across metric quintiles; and made incremental refinements to the scoring approach to optimize metric performance for predicting outcomes across data sets. Refinements involved experimenting with greater or fewer numbers of categories of consumed amounts for use in scoring food groups, exploring different combinations of point values assigned to categories of consumed amounts, and computing total metric scores using subsets of food groups (submetrics) instead of all food groups. Throughout metric evaluation and refinement, we statistically compared the performance of different candidate metrics and submetrics (36 in total) by using Wolfe's tests to compare metric–outcome correlation coefficients (34), and by entering pairs of metrics as predictors in the same regression models and comparing metrics using Wald tests for differences in linear trends (35). Analyses to evaluate and refine metrics were conducted separately using FFQ and 24HR data (when both were available in a given dataset). Where data were available and sample sizes allowed, analyses were conducted both in aggregate and separately by urban compared with rural locality or season of data collection to evaluate robustness of metric performance across contexts and seasons.

### Evaluating performance of final candidate metrics

In the last stage of metric development, we evaluated and compared the performance of the final 3 candidates that we developed [the Global Diet Quality Score (GDQS), GDQS Positive Submetric (GDQS+), and GDQS Negative Submetric (GDQS-)] with a simplified version of the GDQS using fewer categories of consumed amounts for assigning point values, the PDQS-like Metric, and 2 existing metrics. The existing metrics were the Alternative Healthy Eating Index – 2010 (AHEI-2010) (35), which captures diet-related chronic disease risk, and the Minimum Dietary Diversity - Women (MDD-W) indicator (36), which is a proxy for nutrient adequacy (in our analyses, we treated the MDD-W as a continuous variable ranging from 0 to 10, rather than a binary indicator as it is sometimes used) (**Table 2**). Results of cross-sectional analyses reported in this paper focus on associations between metrics and the outcomes they are intended to target by design (i.e., the GDQS+ and MDD-W compared with nutrient adequacy–related outcomes, the GDQS- and AHEI-2010 compared with NCD outcomes, and the GDQS compared with both categories); more expansive results can be found in references 6–10. In addition to analysis of cross-sectional datasets, we analyzed cohort data to evaluate longitudinal associations between change in metrics compared with change in weight and waist circumference, and between metrics and incident type 2 diabetes using the Cox proportional hazards models. In both cross-sectional and cohort data, we also graphically examined nonlinearity in covariate-adjusted metric-outcome relationships to identify GDQS and GDQS submetric cutoffs for defining categorical ranges of diet-related risk for use at the population level.

**TABLE 2:**
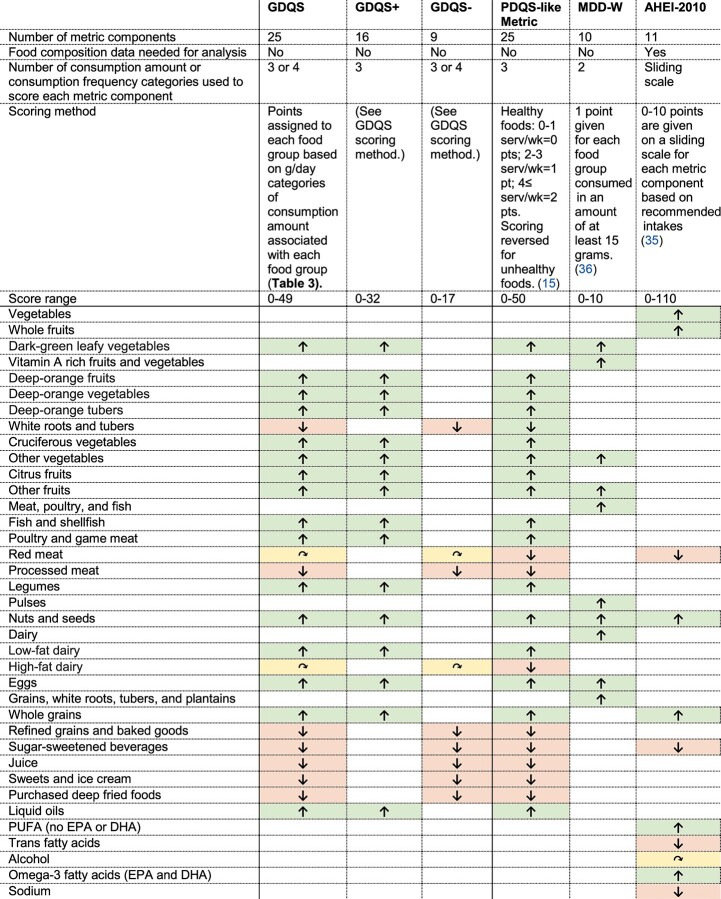
Summary of diet metrics included in the final evaluation^1^

1 Adapted from Fung et al. (15). AHEI-2010, Alternative Healthy Eating Index – 2010; GDQS, Global Diet Quality Score; GDQS+, GDQS Positive Submetric; GDQS-, GDQS Negative Submetric; MDD-W, Minimum Dietary Diversity – Women; PDQS, Prime Diet Quality Score.

Up arrows (green cells) indicate positively scored components (given more points for higher consumption), down arrows (red cells) indicate negatively scored components (given more points for lower consumption), and curved arrows (yellow cells) indicate components for which maximum points are assigned at moderate amounts of consumption. This table excludes the Simplified GDQS (refer to footnote to Table 11 for description), which was also included in the final evaluation.

## Results

The GDQS metric is composed of 25 food groups that are globally important contributors to nutrient intake and/or NCD risk as informed by current nutrition science and epidemiologic literature (16, 17) (**Tables 3** and **4**). Points are assigned based on 3 or 4 categories of consumed amounts (defined in g/d) specific to each group. There are 16 healthy food groups (scored by giving more points for higher intake), 7 unhealthy food groups (more points for lower intake), and 2 food groups classified as unhealthy when consumed in excessive amounts (increasing points are given until specific amounts have been consumed, after which no points are given). The GDQS is obtained by summing points across all of the 25 food groups, ranges from 0 to 49, and is a summary measure of overall diet quality, with respect to both nutrient adequacy and diet-related NCD risk, for use at the population level. GDQS scores ≥23 are associated with a low risk of both nutrient adequacy and NCD risk, scores ≥15 and <23 indicate moderate risk, and scores <15 indicate high risk.

**TABLE 3:** GDQS and GDQS submetric food groups and scoring^1^

Food group	Categories of consumed amounts (g/d)				Point values			
	1	2	3	4	1	2	3	4
Food groups included in the GDQS and GDQS+								
Healthy								
Citrus fruits	<24	24–69	>69		0	1	2	
Deep orange fruits	<25	25–123	>123		0	1	2	
Other fruits	<27	27–107	>107		0	1	2	
Dark green leafy vegetables	<13	13–37	>37		0	2	4	
Cruciferous vegetables	<13	13–36	>36		0	0.25	0.5	
Deep orange vegetables	<9	9–45	>45		0	0.25	0.5	
Other vegetables	<23	23–114	>114		0	0.25	0.5	
Legumes	<9	9–42	>42		0	2	4	
Deep orange tubers	<12	12–63	>63		0	0.25	0.5	
Nuts and seeds	<7	7–13	>13		0	2	4	
Whole grains	<8	8–13	>13		0	1	2	
Liquid oils	<2	2–7.5	>7.5		0	1	2	
Fish and shellfish	<14	14–71	>71		0	1	2	
Poultry and game meat	<16	16–44	>44		0	1	2	
Low fat dairy	<33	33–132	>132		0	1	2	
Eggs	<6	6–32	>32		0	1	2	
Food groups included in the GDQS and GDQS-								
Unhealthy in excessive amounts								
High fat dairy (in milk equivalents)^2^	<35	35–142	>142–734	>734	0	1	2	0
Red meat	<9	9–46	>46		0	1	0	
Unhealthy								
Processed meat	<9	9–30	>30		2	1	0	
Refined grains and baked goods	<7	7–33	>33		2	1	0	
Sweets and ice cream	<13	13–37	>37		2	1	0	
Sugar-sweetened beverages	<57	57–180	>180		2	1	0	
Juice	<36	36–144	>144		2	1	0	
White roots and tubers	<27	27–107	>107		2	1	0	
Purchased deep fried foods	<9	9–45	>45		2	1	0	

1 GDQS, Global Diet Quality Score; GDQS-, GDQS Negative Submetric; GDQS+, GDQS Positive Submetric.

2 Due to the importance of cheese in many food cultures and the significantly different nutrient density of hard cheeses in comparison with other dairy products, we recommend converting consumed masses of hard cheeses to milk equivalents when calculating total consumption of high fat dairy for the purpose of assigning a GDQS consumption category [using cheddar cheese as a typical example, a conversion factor of 6.1 can be computed as the mass of 1 serving of milk (237 mL × 0.95 g/mL = 225 g) divided by an isocaloric mass of cheddar cheese (37 g)] (38).

**TABLE 4:** Description of the GDQS food groups^1^

Food group	Description
Citrus fruits	Whole fruits in the genus *Citrus*
Deep orange fruits	Whole fruits (not including juice or spreads) containing ≥20 retinol equivalents/100 g
Other fruits	Whole fruits not belonging in the other fruit categories (not including coconuts)
Dark green leafy vegetables	Leafy vegetables containing ³120 retinol equivalents/100 g
Cruciferous vegetables	Vegetables in the family *Brassicaceae*
Deep orange vegetables	Nontuberous vegetables containing ≥120 retinol equivalents/100 g
Other vegetables	Vegetables not belonging in the other vegetable categories
Legumes	Legumes and foods derived from legumes, such as tofu and soymilk. Does not include bean sprouts (classified in “Other vegetables”) or groundnuts (classified in “Nuts and seeds”)
Deep orange tubers	Tuberous vegetables containing ≥120 retinol equivalents/100 g (includes variants biofortified with vitamin A)
Nuts and seeds	Nuts, seeds, and products derived from nuts and seeds, such as nut-based butters (but not oils). Also includes groundnuts. Seeds that are used as spices are included when used in their whole (not powdered) form
Whole grains	Whole grains and whole-grain products. Does not include products with significant amounts of added sugar (classified as “Sweets and ice cream”)
Liquid oils	All types of oils that are liquid at room temperature, regardless of fatty acid profile (this includes palm olein, liquid palm kernel oil, and liquid coconut oil). Does not include oil used to deep fry foods that are purchased, but does include oil used to deep-fry foods prepared at home
Fish and shellfish	Fish (whether processed or unprocessed) based on phylogenetic classifications (including sharks, eels, and rays), and other seafood high in n3 fatty acids (including shellfish, jellyfish, cetaceans, and pinnipeds, but not echinoderms). Includes organs
Poultry and game meat	Unprocessed poultry and game, including a range of undomesticated animals and bush meat, e.g., primates, rodents, canines, felines, marsupials, leporids (rabbits and hares), wild boar, bats, bears, semiaquatic mammals (including otters and beavers), undomesticated ungulates, reptiles (aquatic and terrestrial), and amphibians. Includes organs
Low fat dairy	Reduced or naturally low fat dairy products (≤2% milk fat). Includes flavored milk, and milk or cream added to coffee or tea
Eggs	All types of eggs. Does not include mayonnaise
High fat dairy	High fat milk and dairy products (>2% milk fat). Includes flavored milk, and milk or cream added to coffee or tea. Does not include butter or clarified butter. This category also does not include ice cream and whipped cream
Red meat	Unprocessed red meat belonging to domesticated animals (i.e., not game), including organs. “Red” classification is not based on color but on nutritional characteristics, and thus includes pork and lamb
Processed meat	Processed red meat, poultry, or game, including organs, and excluding fish and seafood. Processing is defined per International Agency for Research on Cancer: “salting, curing, fermentation, smoking or other processes to enhance flavor or improve preservation.”
Refined grains and baked goods	Refined grains and refined grain products. Does not include products with significant amounts of added sugar, which should instead be classified as “Sweets and ice cream”
Sweets and ice cream	Sugar-sweetened foods that are not beverages; includes sugar and other caloric sweeteners added to other foods and drinks. Whipped cream also classified in this category
Sugar-sweetened beverages	Sweetened drinks that do not contain any fruit juice at all. Includes, e.g., sodas, energy drinks, sports drinks, and beverages made using low-calorie sweeteners, such as diet sodas. Sweetened tea and coffee, and dairy or cereal-based drinks are not included
Juice	Unsweetened or sweetened drinks that are at least partly composed of fruit juice. This category also includes fruit smoothies made from whole fruit
White roots and tubers	Tuberous vegetables with <120 retinol equivalents/100 g. Includes flours such as potato or cassava flour
Purchased deep fried foods	Deep fried foods fried in an amount of fat or oil sufficient to cover the food completely. Only deep fried foods that are purchased (i.e., not prepared at home) are classified in this group. Foods in this category are “double classified” and should be classified as belonging to the purchased deep fried food group as well as the food group to which the food normally belongs if not purchased and deep fried (e.g., deep fried white potatoes that are purchased should be classified in both the purchased deep fried foods group and in the white roots and tubers group).

1 Semisolid and solid fats and insects are excluded from GDQS scoring. Coconuts and coconut products (e.g., coconut milk) are also excluded (the exception is liquid coconut oil, which is included in the liquid oils group). The following beverages are also excluded from GDQS scoring: alcohol, coffee, and tea. However, if milk is added to coffee or tea, the added milk should be classified in the high or low fat dairy food group, and if a caloric sweetener (e.g., sugar) is added to coffee or tea, the caloric sweetener should be classified in the sweets and ice cream food group. As a simple metric of diet quality, the GDQS does not intend to capture information related to the consumption of nutrient fortificants; fortified foods should be classified in the food group that corresponds to the unfortified version of that food (e.g., orange juice fortified with calcium should be classified in the juice category, liquid oil fortified with vitamin A should be classified in the liquid oil category, etc.). GDQS, Global Diet Quality Score.

The GDQS+ submetric includes the 16 healthy food groups included in the GDQS, is scored with the same categories of consumed amounts used in the GDQS, and ranges from 0 to 32. The GDQS- submetric includes the 9 GDQS food groups classified as unhealthy or unhealthy in excessive amounts, is scored with the same categories of consumed amounts used in the GDQS, and ranges from 0 to 17. The GDQS+ and GDQS- quantify the collective contribution of healthy foods (those that should be consumed in higher amounts) and unhealthy foods (those that should be consumed in lower amounts), respectively, to overall diet quality (because higher consumption of red meat and high fat dairy are scored as unhealthy, these food groups are included in the GDQS-). The GDQS submetrics can be further subdivided to give more detailed information about the contribution of smaller sets of food groups or individual food groups to diet quality in populations. We did not find evidence to support ranges of GDQS+ or GDQS- scores for defining categories of dietary risk.

### Associations between the GDQS and comparison metrics compared with energy-adjusted nutrient intakes and overall nutrient adequacy in cross-sectional datasets

In analysis of cross-sectional data, we observed Spearman correlations between the GDQS and energy-adjusted intakes of calcium, fiber, folate, iron, protein, vitamin A, and zinc that were generally modest and weaker and in some cases had inverse associations with specific fatty acids and vitamin B12 (**Table 5**). The GDQS tended to correlate more favorably (*P* < 0.05) than the MDD-W with energy-adjusted fiber, folate, iron, protein, saturated fat, and zinc intakes, whereas the MDD-W tended to correlate better with energy-adjusted monounsaturated fat, vitamin A, and vitamin B12 intakes.

**TABLE 5:**
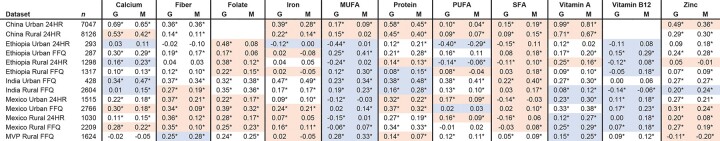
Comparison of Spearman correlations between the GDQS and MDD-W compared with energy-adjusted nutrients among nonpregnant nonlactating women of reproductive age within urban and rural strata of cross-sectional datasets^1^

1 Values are Spearman correlations unless otherwise indicated. *P < 0.05. Red shading indicates ρ_GDQS_ > ρ_MDD−W_ (Wolfe's test *P* for difference < 0.05) and blue shading indicates ρ_GDQS_ < ρ_MDD−W_. Shading is reversed for saturated fat. G/GDQS, Global Diet Quality Score; M/MDD-W, Minimum Dietary Diversity – Women; MVP, Millennium Villages Project; 24HR, 24-hour recall.

**FIGURE 1:**
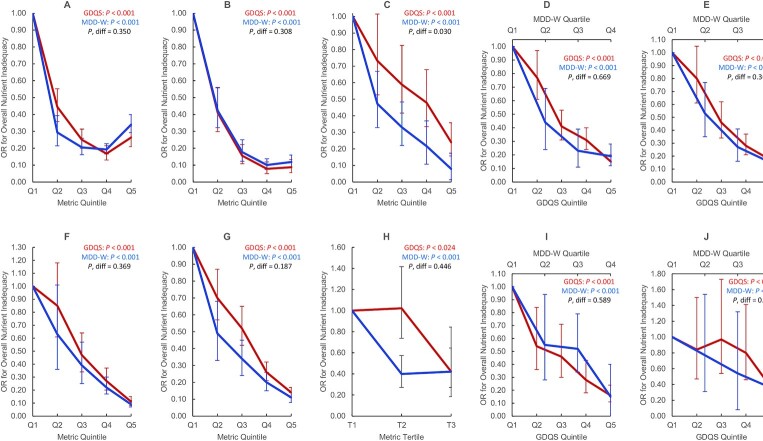
Covariate-adjusted ORs for binary overall nutrient inadequacy by GDQS and MDD-W quintile, quartile, or tertile in nonpregnant nonlactating women of reproductive age in the total population or within urban stratum and rural strata of cross-sectional datasets. We defined several aggregate measures of protein, fiber, calcium, iron, zinc, vitamin A, folate, and vitamin B12 adequacy. In FFQ analysis, a continuous overall nutrient adequacy variable was first constructed for each participant in the data, based on the number of nutrients (out of 8) meeting age- and sex-specific EARs from the Institute of Medicine (or adequate intake level, in the case of fiber) (39); iron adequacy was defined as ≥50% probability of adequacy based on a lognormal requirement distribution (40). In 24HR analysis [based on 3-day averages (China) or estimated usual intakes based on the ISU method (Mexico) (41)], probability of adequacy for all nutrients was estimated using the full probability method (40). Iron requirement distributions and zinc EARs were adjusted to account for absorption characteristics of local diets (40,42–44). Because nutrient requirements are age-specific, they indirectly account for age differences in energy intake to an extent, but not entirely. To account for residual confounding by energy, we therefore adjusted overall nutrient adequacy for energy using the residual method (45), and added the resulting residuals back to the mean of the raw overall nutrient adequacy variable. We derived a binary measure of overall nutrient inadequacy defined as <4 adequate nutrients (in FFQ data) or < 50% mean probability of adequacy (in 24HR data). We also derived a binary measure of energy-adjusted overall nutrient inadequacy (shown in this figure) by adjusting the continuous overall nutrient adequacy variable for energy using the residual method, ranking the residuals, and assigning a value of 1 to those in the top Xth percentile and 0 to those in the bottom, in which X is the proportion of individuals in the raw data with <4 adequate nutrients (in FFQ data) or <50% mean probability of adequacy (in 24HR data). Energy-adjusted overall nutrient inadequacy therefore preserves the distribution of raw overall nutrient inadequacy. This figure displays linear trends in overall nutrient inadequacy across metric quintiles (*P*), statistically compared using regression models in which quintiles of 2 metrics are included in the same model and the parameter estimates associated with quintile 5 are compared using a Wald test (*P*, diff) (35). Models were adjusted for age (India and Millennium Villages); age, urban/rural locality, education, marital status, occupation (Ethiopia); age, socioeconomic status, education, physical activity, smoking, alcohol use, occupation, urban/rural locality (China); age, socioeconomic status, urban/rural locality (Mexico). Trends did not differ between GDQS and MDD-W, except in analysis of Ethiopia FFQ data (in which the MDD-W was more predictive). Due to limited variation across metric quintiles, MDD-W is presented in terms of quartiles in Mexico FFQ and 24HR data, and tertiles in Ethiopia 24HR data. India Total Population FFQ (*n =* 3065) (A), Millennium Villages Project Rural FFQ (*n =* 1624) (B), Ethiopia Total Population FFQ (*n =* 1604) (C), Mexico Urban FFQ (*n =* 2766) (D), Mexico Rural FFQ (*n =* 2209) (E), China Urban 24HR (*n =* 7047) (F), China Rural 24HR (*n =* 8126) (G), Ethiopia Total Population 24HR (*n =* 1593) (H), Mexico Urban 24HR (*n =* 1515) (I), Mexico Rural 24HR (*n =* 1030) (J). EAR, estimated average requirement; GDQS, Global Diet Quality Score; MDD-W, Minimum Dietary Diversity – Women; 24HR, 24-h recall.

We also compared covariate-adjusted associations between metrics and energy-adjusted aggregate measures of protein, fiber, calcium, iron, zinc, vitamin A, folate, and vitamin B12 adequacy (refer to footnote to **Figure 1** for derivation of these measures and adjustment covariates). In one dataset (Ethiopia FFQ data), the MDD-W outperformed the GDQS (*P* < 0.05) in predicting overall nutrient inadequacy (a binary variable defined as adequacy of <4 out of 8 nutrients in FFQ analysis or <50% mean probability of adequacy in 24HR analysis) in adjusted models: OR in the fifth quintile (compared with quintile 1) of the GDQS and MDD-W was 0.24 (95% CI: 0.16, 0.36) and 0.08 (95% CI: 0.08, 0.14), respectively (Figure 1) (9). Performance of the GDQS and MDD-W in predicting overall nutrient inadequacy did not otherwise differ. The GDQS and MDD-W tended to correlate more strongly with energy-adjusted intakes of nutrient intakes and adequacy than the GDQS+ and AHEI-2010, and the GDQS- tended to correlate weakly or negatively (6–10).

### Covariate-adjusted associations between the GDQS, GDQS+, and MDD-W compared with anthropometric and biomarker outcomes related to nutrient adequacy in cross-sectional datasets

The GDQS and GDQS+ performed comparably with the MDD-W in predicting anthropometric and clinical indicators of nutrient adequacy in cross-sectional analyses. In adjusted regression models (refer to footnote to Figure 1 for adjustment covariates), the GDQS, GDQS+, and MDD-W were significantly (*P* for trend < 0.05) inversely associated with underweight [BMI (kg/m^2^) <18.5] in Ethiopia FFQ data and India; low mid–upper arm circumference (<24.5 cm) in Ethiopia FFQ data, India, and the Millennium Villages; and unassociated with underweight or anemia in urban or rural China (**Table 6**). These metrics were also inversely associated with serum folate deficiency (<3 ng/mL) in Ethiopia FFQ data, associated with higher serum folate concentrations in 24HR data from urban Mexico, inversely associated with anemia (hemoglobin <12 g/dL) in the African Millennium Villages, and associated with higher hemoglobin concentrations or inversely associated with anemia in Ethiopia FFQ data (**Table 7**).

**TABLE 6:**
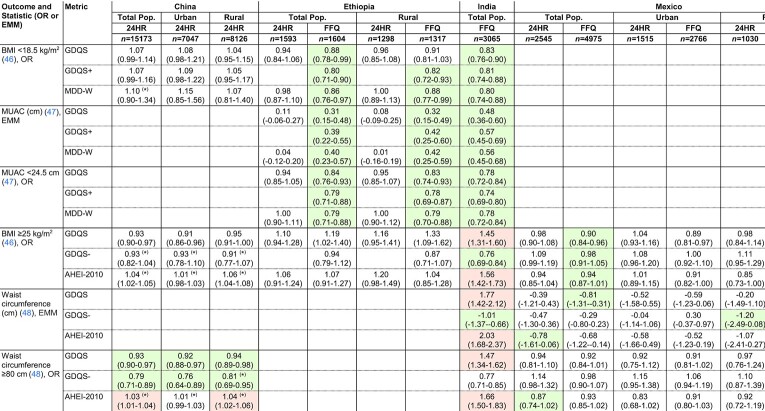
Covariate-adjusted associations between metrics and anthropometric outcomes among NP NL women of reproductive age in the total population or within the urban or rural stratum of cross-sectional datasets^1^

1 Values indicate ORs or EMMs (95% CIs) per 1-SD increase in metrics. ORs and EMMs are estimated from covariate-adjusted regression models of associations between metrics (expressed in quintiles) and continuous outcomes, or dichotomous outcomes defined according to clinically relevant cutoffs. See footnote to Figure 1 for adjustment covariates. Color indicates statistically significant linear trend across metric quintiles (*P* < 0.05) (green, protective; red, deleterious). *P < 0.05, statistically significant Wald test comparing trends between the GDQS and other metrics. Sample size corresponds to the number of participants with dietary data [for some outcomes, available sample size was smaller; refer to (25–33) for more details]. AHEI-2010, Alternative Healthy Eating Index – 2010; EMM, Estimated Marginal Mean; GDQS, Global Diet Quality Score; GDQS+, GDQS Positive Submetric; GDQS-, GDQS Negative Submetric; MDD-W, Minimum Dietary Diversity – Women; MUAC, mid–upper arm circumference; MVP, Millennium Villages Project; 24HR, 24-hour recall.

**TABLE 7:**
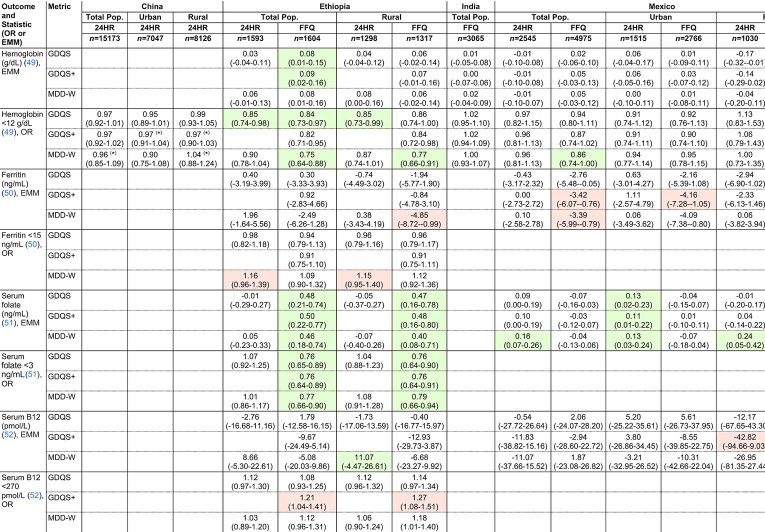
Covariate-adjusted associations between the GDQS, GDQS+, and MDD-W compared with biochemical outcomes related to nutrient adequacy among nonpregnant nonlactating women of reproductive age in the total population or within urban stratum and rural strata of cross-sectional datasets^1^

1 Values presented as ORs or estimated marginal means (EMM) (95% CI) per 1-SD increase in metrics. ORs and EMMs are estimated from covariate-adjusted regression models of associations between metrics (expressed in quintiles) and continuous outcomes, or dichotomous outcomes defined according to clinically relevant cutoffs. See footnote to Figure 1 for adjustment covariates. Color indicates statistically-significant linear trend across metric quintiles (*P* < 0.05) (green: protective, red: deleterious). *P < 0.05 for Wald test comparing trends between the GDQS and other metrics. Sample size corresponds to the number of participants with dietary data [for some outcomes, available sample size was smaller; refer to (25–33) for more details]. EMM. estimated marginal mean; GDQS, Global Diet Quality Score; MDD-W, Minimum Dietary Diversity – Women; MVP, Millennium Villages Project; 24HR, 24-hour recall.

Unlike the GDQS, the MDD-W was associated with higher odds of depleted iron stores (serum ferritin <15 μg/L) in Ethiopia 24HR data (OR for tercile 3 compared with T1: 2.68, 95% CI: 1.35, 5.20) (7), and both the MDD-W and GDQS+ predicted lower ferritin concentrations in Mexico FFQ data (8) (Table 7). The GDQS+ was further associated with higher odds of serum vitamin B12 deficiency (<203 pg/mL) in Ethiopia FFQ data (OR for quintile 5 compared with quintile 1: 1.83, 95% CI: 1.14, 2.98) and inversely associated with vitamin B12 concentrations in 24HR data from rural Mexico (quintile 1 compared with quintile 5 difference in estimated marginal mean: 646 compared with 428 pmol/l) (7, 8).

### Covariate-adjusted associations between the GDQS, GDQS-, and AHEI-2010 compared with the metabolic syndrome and anthropometric and biomarker outcomes related to NCD risk in cross-sectional datasets

The GDQS significantly outperformed (*P* < 0.05) the AHEI-2010 in predicting the metabolic syndrome (MetS; defined according to ATP III criteria) in urban China: OR for MetS in the fifth quintile of GDQS (compared with quintile 1) was 0.58 (95% CI: 0.45, 0.75) (9), whereas the AHEI-2010 was not significantly predictive (*P* = 0.63) (**Table 8**). In rural China, the AHEI-2010 was positively associated with MetS (fifth quintile OR compared with quintile 1 1.32, 95% CI: 1.06, 1.63) (9), whereas the GDQS was marginally associated with lower odds of MetS (*P* = 0.054) and significantly associated with lower odds of high waist circumference (≥80 cm), hypertension (>130/85 mmHg), and low HDL cholesterol (<50 mg/dL) (Table 6, Table 8, **Table 9**). Both the GDQS and AHEI-2010 were positively associated with overweight (BMI ≥25) and high waist circumference in India (Table 6). Although not predictive of the MetS in urban or rural Mexico in 24HR or FFQ analyses (Table 8), the GDQS was inversely associated with continuous BMI, waist circumference, and LDL cholesterol in FFQ analysis (8).

**TABLE 8:**
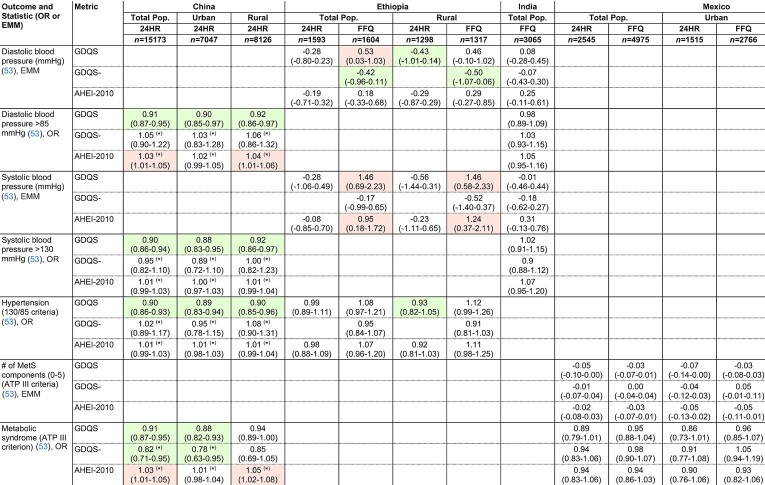
Covariate-adjusted associations between the GDQS, GDQS-, and AHEI-2010 compared with blood pressure and MetS among nonpregnant nonlactating women of reproductive age in the total population or within urban stratum and rural strata of cross-sectional datasets^1^

1 Values indicate OR or EMM (95% CI) per 1-SD increase in metrics. ORs and EMMs estimated from covariate-adjusted regression models of associations between metrics (expressed in quintiles) and continuous outcomes, or dichotomous outcomes defined according to clinically relevant cutoffs. See footnote to Figure 1 for adjustment covariates. Color indicates statistically significant linear trend across metric quintiles (*P* < 0.05) (green, protective; red, deleterious). *P < 0.05 for Wald test comparing trends between the GDQS and other metrics. Sample size corresponds to the number of participants with dietary data [for some outcomes, available sample size was smaller; refer to (25–33) for more details]. AHEI-2010, Alternative Healthy Eating Index – 2010; ATP, Adult Treatment Panel; EMM. estimated marginal mean; GDQS, Global Diet Quality Score, MetS, metabolic syndromel 24HR, 24-hour recall.

**TABLE 9:**
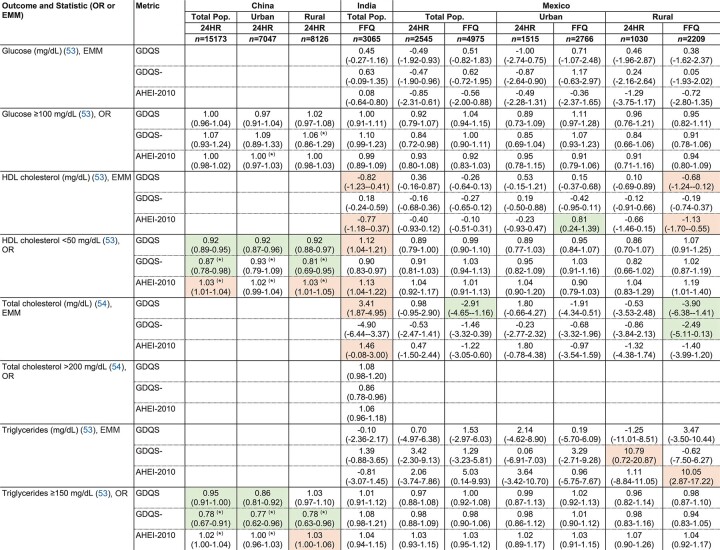
Covariate-adjusted associations between the GDQS, GDQS-, and AHEI-2010 compared with biochemical outcomes related to NCD risk among nonpregnant nonlactating women of reproductive age in the total population or within urban and rural strata of cross-sectional datasets^1^

1 Values indicate ORs or EMM (95% CI) per 1SD increase in metrics. ORs and EMMs are estimated from covariate-adjusted regression models of associations between metrics (expressed in quintiles) and continuous outcomes, or dichotomous outcomes defined according to clinically relevant cutoffs. See footnote to Figure 1 for adjustment covariates. Color indicates statistically-significant linear trend across metric quintiles (*P* < 0.05) (green: protective, red: deleterious). *P < 0.05 Wald test comparing trends between the GDQS and other metrics. Sample size corresponds to the number of participants with dietary data (for some outcomes, available sample size was smaller; refer to (25–33) for more details). AHEI-2010, Alternative Healthy Eating Index – 2010; GDQS, Global Diet Quality Score; 24HR, 24-hour recall.

Like the GDQS, the GDQS- (of which higher scores indicate lower consumption of unhealthy foods) was significantly inversely associated with MetS in urban China, low HDL cholesterol in rural China, and high waist circumference and triglycerides (≥150 mg/dL) in both urban and rural China (Table 6, Table 8, Table 9). The GDQS- was also associated with lower waist circumference and odds of overweight in India, negatively associated with diastolic blood pressure in Ethiopia FFQ data, and negatively associated with total cholesterol in FFQ data from rural Mexico (Table 6, Table 8, Table 9). Although the GDQS- was not significantly associated with the MetS in rural Mexico (*P* = 0.85 and *P* = 0.82 in 24HR and FFQ analyses, respectively), it was associated with having a reduced number of MetS components in FFQ analysis (Table 8) (quintile 1 compared with quintile 5 difference in estimated marginal mean number of components: 2.54 compared with 2.35) (8).

### Longitudinal analysis of cohort datasets

In multivariable analysis of women in the Mexican Teachers’ Cohort, a 1-SD increase in GDQS over 2 y was associated with 0.37 kg (95% CI: 0.27, 0.47) less gain in weight (AHEI-2010: 0.33 kg, 95% CI: 0.22, 0.44; MDD-W kg: 0.26 95% CI: 0.14, 0.37) and 0.52 cm (95% CI: 0.33, 0.71) less gain in waist circumference (AHEI-2010: 0.24 cm, 95% CI: 0.03, 0.45; MDD-W: -0.42 cm, 95% CI: 0.20, 0.63) (11). The GDQS was significantly (*P* < 0.05) more strongly associated with weight change than the MDD-W and with waist circumference change than the AHEI-2010.

In multivariable analysis of women in the US Nurses’ Health Study II (NHS II) cohort, each 1-SD increase in GDQS and AHEI-2010 over 4 y was associated with an HR for 5-kg weight gain of 0.86 (95% CI: 0.85, 0.87) and 0.80 (95% CI: 0.80, 0.81), respectively, whereas each 1 SD increase in GDQS and AHEI-2010 was associated with a HR for type 2 diabetes of 0.93 (95% CI: 0.91, 0.96) and 0.91 (95% CI: 0.88, 0.94), respectively, (*P* < 0.05 for difference in metrics for both outcomes) (12, 13). A 1-SD increase in the MDD-W was significantly (*P* < 0.05) less predictive than the GDQS of 5-kg weight gain (HR: 0.95, 95% CI: 0.94, 0.95), and did not predict incident diabetes (HR: 1.00, 95% CI: 0.94, 1.04, *P* = 0.88). In the NHS II, 4-y increases in the GDQS- submetric were also particularly predictive of weight change (HR for a 1 SD increase in GDQS-: 0.82, 95% CI: 0.81, 0.82) and incident diabetes (HR associated with fifth quintile of GDQS- compared with Q1: 0.76, 95% CI: 0.69, 0.84; for comparison, the HR associated with the fifth quintile of GDQS compared with quintile 1: 0.83, 95% CI: 0.76, 0.91).

A comparison of changes in weight and waist circumference observed for different magnitudes of change in the GDQS, GDQS+, and GDQS- in the 2 cohort studies is provided in **Table 10**.

**TABLE 10:** Covariate-adjusted associations between change in GDQS, GDQS+, and GDQS- compared with change in weight and waist circumference among women <50 years of age in the Mexican Teachers’ Cohort and US Nurses’ Health Study II

Dataset outcome metric	Large decrease	Small decrease	Little change	Small increase	Large increase
Mexican Teachers’ Cohort					
2-y weight change (kg)					
GDQS	< −5 pts: 0.50 (0.19, 0.81)	−5 to < −2 pts: 0.33 (0.09, 0.57)	−2 to 2 pts: (Ref)	>2 to 5 pts: −0.43 (−0.67, −0.20)	>5 pts: −0.81 (−1.11, −0.51)
GDQS+	< −2.7 pts: 0.22 (−0.04, 0.50)	−2.7 to < −.2 pts: 0.10 (−0.16, 0.36)	0 to 1.7 pts: (Ref)	>2.0 to 4.0 pts: −0.21 (−0.47, 0.05)	>4.2 pts: -0.52 (−0.79, −0.24)
GDQS-	< −2.0 pts: 0.36 (0.10, 0.62)	−2.0 to < −1.0 pts: 0.14 (−0.14, 0.42)	0 pts: (Ref)	>1 to 1 pts: −0.20 (−0.48, 0.07)	>2 pts: −0.25 (−0.51, 0.01)
2-y waist circumference change (cm)					
GDQS	< −5 pts: 0.71 (0.09, 1.32)	−5 to < −2 pts: 0.32 (−0.12, 0.77)	−2 to 2 pts: (Ref)	>2 to 5 pts: −0.49 (−0.94, −0.04)	>5 pts: −1.05 (−1.62, −0.48)
GDQS+	< −2.7 pts: 0.32 (−0.19, 0.84)	−2.0 to < −0.2 pts: 0.10 (−0.41, 0.61)	0 to 1.7 pts: (Ref)	>2.0 to 4.0 pts: −0.22 (−0.73, 0.28)	>4.2 pts: -0.79 (−1.32, −0.27)
GDQS-	< −2.0 pts: 0.98 (0.47, 1.48)	−2.0 to < −1.0 pts: 0.80 (0.25, 1.34)	0 pts: (Ref)	>1 to 1 pts: 0.49 (−0.04, 1.03)	>2 pts: -0.07 (−0.57, 0.43)
US Nurses' Health Study II					
4-y weight change (kg)					
GDQS	< −5 pts: 1.13 (1.04, 1.22)	−5 to < −2 pts: 0.45 (0.38, 0.53)	−2 to 2 pts: (Ref)	>2 to 5 pts: −0.49 (−0.56, −0.42)	>5 pts: -1.24 (−1.31, −1.16)
GDQS+	< −5 pts: 0.52 (0.43, 0.62)	−5 to < −2 pts: 0.27 (0.20, 0.34)	−2 to 2 pts: (Ref)	>2 to 5 pts: −0.27 (−0.33, −0.20)	>5 pts: −0.56 (−0.64, −0.48)
GDQS-		< −2 pts: 1.19 (1.12, 1.27)	−2 to 2 pts: (Ref)	>2 pts: −1.29 (−1.36, −1.23)	

1 Values represent point change in metric: change in weight (kg) or waist circumference (cm) (95% CI for change). Mexican Teachers’ Cohort analysis (11) is adjusted for state (Jalisco or Veracruz), marital status, education, and health insurance type; baseline diet metric score, age, BMI category, and asset score; pre- and post–physical activity level; and changes in total energy intake, smoking status, and alcohol use. Nurses’ Health Study II analysis (12) is adjusted for age, time period, sleep duration, and oral contraceptive use; baseline metric score; and changes in physical activity level, sitting, smoking status, and alcohol use. GDQS, Global Diet Quality Score; GDQS+, GDQS Positive Submetric; GDQS-, GDQS Negative Submetric.

### Evaluating the PDQS-like Metric and Simplified GDQS

A simplified version of the GDQS (employing fewer categories of consumption amounts used to score food groups) tended to exhibit less predictive value, particularly against overall nutrient adequacy (**Table 11**). The GDQS also tended to outperform the PDQS-like Metric in this respect (Table 11) and in predicting MetS in China (9). In urban China, ORs for MetS in the fifth quintile of the GDQS and PDQS-like Metric (compared with quintile 1) were 0.58 (95% CI: 0.45, 0.75, *P* for trend < 0.001) and 0.86 (95% CI: 0.69, 1.08, *P* = 0.224), respectively (*P* for difference < 0.05) (9). In rural China, ORs for MetS in the fifth quintile of the GDQS and PDQS-like Metric (compared with Q1) were 0.87 (95% CI: 0.69, 0.1.10, *P* for trend = 0.054) and 1.01 (95% CI: 0.81, 1.26, *P* = 0.943), respectively (*P* for difference < 0.05) (9).

**TABLE 11:** Comparison of Spearman correlations between the GDQS, simplified GDQS, and PDQS-like metric and continuous energy-adjusted overall nutrient adequacy in nonpregnant nonlactating women of reproductive age within urban and rural strata of cross-sectional datasets

		GDQS	Simplified GDQS		PDQS-like Metric	
Dataset	*n*	ρ	ρ	*P*, diff	ρ	*P*, diff
China Urban 24HR	6902	0.42*	0.32*	<0.001*	0.35*	<0.001*
China Rural 24HR	8036	0.31*	0.25*	<0.001*	0.23*	<0.001*
Ethiopia Urban 24HR	285	0.02	0.02	0.781	−0.06	0.011*
Ethiopia Urban FFQ	285	0.32*	0.25*	0.006*	0.16*	0.029*
Ethiopia Rural 24HR	1283	0.08*	0.09*	0.013*	−0.07*	<0.001*
Ethiopia Rural FFQ	1311	0.25*	0.21*	0.002*	0.11*	0.000*
India Urban FFQ	428	0.13*	0.13*	0.040*	0.14*	0.933
India Rural FFQ	2600	0.32*	0.31*	0.817	0.21*	<0.001*
Mexico Urban 24HR	1464	0.32*	0.27*	<0.001*	0.21*	<0.001*
Mexico Urban FFQ	2696	0.40*	0.34*	<0.001*	0.28*	<0.001*
Mexico Rural 24HR	1003	0.22*	0.19*	0.010*	0.10*	<0.001*
Mexico Rural FFQ	2175	0.35*	0.30*	<0.001*	0.19*	<0.001*
MVP Rural FFQ	1624	0.37*	0.36*	0.790	0.31*	<0.001*

The Simplified GDQS was generated by condensing the second and third consumption amount categories in Table 3 for all food groups (except red meat, for which trichotomous scoring was retained to allow for healthy scoring at higher intake amounts, as in the GDQS). Refer to footnote to Figure 1 for derivation of the continuous energy-adjusted overall nutrient adequacy variable. *P* for difference (*P*, diff) is estimated from using Wolfe's tests comparing metric-outcome correlation coefficients between the GDQS and either the Simplified GDQS or PDQS-like Metric (34). *P < 0.05 correlations and Wolfe's tests. GDQS, Global Diet Quality Score; MVP, Millennium Villages Project; PDQS, Prime Diet Quality Score; 24HR, 24-hour recall.

## Discussion

Using analysis of existing datasets from multiple countries representing a wide range of income levels and cultures, we found that a simple, food-based GDQS captured both nutrient adequacy and diet-related risk of NCDs and performed comparably with existing metrics that have been developed for more specific applications or populations. To our knowledge, the GDQS is the first food-based metric of diet quality to be comprehensively validated against health outcomes representative of both of these key domains of malnutrition in diverse regions.

The GDQS performed well compared with the MDD-W in capturing nutrient adequacy, and anthropometric and biochemical indicators of undernutrition. Comparable (and in 1 instance superior) performance of the MDD-W in predicating overall nutrient adequacy is not surprising since unlike the GDQS, the MDD-W scores all foods positively, and unhealthy foods can contribute to nutrient adequacy. Furthermore, although modest consumption of red meat and high fat dairy are scored positively in the GDQS, high consumption is not, and women with the highest intakes of these groups therefore received lower GDQS scores. The GDQS’ inclusion of unhealthy food groups and foods scored as unhealthy in excessive amounts was important for the metric's ability to capture NCD outcomes, particularly in analysis of cohort data from Mexico and the US. Given that inclusion of unhealthy foods did not significantly compromise the GDQS’ ability to capture nutrient adequacy-related outcomes, the GDQS presents a favorable alterative to the MDD-W that, despite its simplicity, is not designed to address NCD risk (an increasingly important form of malnutrition in LMIC).

The GDQS also performed comparably or better than the AHEI-2010 in capturing diet-related NCD risk. This suggests that the GDQS’ expanded food list (as compared with other food-based metrics, such as the MDD-W) compensates for the predictive advantage that might otherwise be gained by including nutrients in metric scoring. Strong performance of the GDQS against NCD outcomes is important given the relative ease of scoring the GDQS (a food-based metric) in comparison with the AHEI-2010, which requires food composition data to score, and which was not as sensitive to nutrient adequacy outcomes in this analysis.

Throughout the process of refining the GDQS and iteratively evaluating its performance, we found that including both healthy- and unhealthy-scoring food groups and scoring each of them in a consistent manner independent of the outcome being targeted generally strengthened associations with both nutrient adequacy and NCD risk-related outcomes. This can be explained by the fact that consumption of healthy, nutrient-dense foods may serve to replace unhealthy foods in the diet, or contribute directly to improved metabolic health [for example, consumption of fruits and vegetables is associated with lower blood pressure, lower blood sugar, weight loss, and reduced incidence of cardiovascular disease, certain cancers, and other diseases (55–58)]; conversely, consumption of unhealthy foods may contribute to poorer nutrient adequacy by replacing healthy foods. Thus, although the GDQS+ and GDQS- outperformed the GDQS in certain cases, we propose the GDQS as the most appropriate and broadly responsive metric of diet quality. The responsiveness of the GDQS to both nutrient adequacy and NCD risk further substantiates construction and scoring of the submetrics and supports their use for understanding and tracking the relative contributions of healthy and unhealthy food groups to overall diet quality in populations.

In many cases, the GDQS was more sensitive to outcomes than a simplified version primarily employing dichotomous categories for scoring consumed amounts. Based on this, we suggest there is a benefit in using diet quality metrics that incorporate more detailed quantity of consumption information than has previously been the case for simple food group indicators developed for use in LMIC, such as the MDD-W, despite the added burden for data collection that is implied. However, more detailed evaluations of this tradeoff between predictive value and participant burden are needed.

An important consideration is that our primary objective was to develop a metric to assess average diet quality for a population or subpopulation. When calculated from a single 24-h recall of food intake, the GDQS will not provide appropriate data on distributions of diet quality (for this reason performance of the GDQS and other metrics tabulated using 24HR data in Ethiopia and Mexico was usually poorer than when tabulated using the FFQ in the same settings). To accurately estimate the distribution of usual intakes or associations between the GDQS with health outcomes at the individual level, calculating the GDQS using an FFQ or repeated 24HRs or diet records in at least a subgroup will be needed.

An important strength of the GDQS development work carried out to date is its inclusion of datasets from a range of countries with different prevailing diet patterns, profiles of disease burden, and levels of economic development. The scope of our analysis was considerable, including a range of nutrient adequacy and diet-related NCD outcomes to develop novel metrics and characterize their validity and flexibility, and disaggregation of datasets to compare metric validity across urban and rural subgroups. Analysis of both FFQ and 24HR data allowed comparison of the performance of metrics scored using both types of instruments. Furthermore, inclusion of cohort data from the US and Mexico allowed more powerful evaluation of metric performance, particularly in capturing NCD-related outcomes (examining cross-sectional associations between diet and NCD outcomes is challenging given the latency period between dietary exposures and such outcomes, and the potential for reverse causation).

The GDQS’ food-based design is a notable strength of the metric. Analysis is simplified because food composition data are not required to analyze the GDQS, and data collection is therefore simplified because detailed information on food preparation methods need not be collected. This ease of use enables rapid and time-relevant assessments of population diet quality. Since many LMIC face a chronic lack of resources for conducting detailed diet surveys and maintaining up-to-date food composition data, the GDQS’ food-based design and ease of use are also conducive to cross-country comparisons. Furthermore, because the GDQS and GDQS submetrics provide information on the contribution of healthy and unhealthy food groups to diet quality in populations, they provide more easily communicated and actionable data for improving diet quality than metrics scored using nutrient components.

A limitation of this research is that it did not include primary data collection. Whereas secondary analysis allowed for extensive data to develop and evaluate metrics, metrics scored using existing dietary data do not exactly represent what would be collected using stand-alone instruments designed to assess these metrics directly. Although existing 24HR and FFQ data have the benefit of extensive enumeration of foods with which to score metrics, they are subject to different sources of measurement error than a stand-alone GDQS tool would be, including greater respondent burden owing to the more extensive assessment. Carrying out primary validation studies of stand-alone GDQS assessment methods in a variety of settings and demographic groups will be a useful next step. On the other hand, the existing datasets did demonstrate that the GDQS can be usefully calculated from quantitative 24HRs and FFQs that are already being used in many countries [we have published detailed guidance for investigators interested in doing this (37)]. Elsewhere in this supplement, Moursi and colleagues describe development of a novel application–based 24HR data collection system, combining a software application with physical food group quantity models, for collecting GDQS data in population surveys (14).

A second limitation is that within each food group there exists regional variation in foods (e.g., lean pasture-fed beef compared with beef produced in concentrated animal feeding operations), food processing methods (e.g., application or absence of micronutrient fortificants in grain flours), and susceptibility of populations to health effects of different diets (e.g., the marginal risk of NCD outcomes incurred by consuming unhealthy foods may be greater in obese or diabetic populations than healthy ones). Although these considerations could be addressed by adding complexity to the scoring system (e.g., by adding or subdividing food groups, or tailoring scoring weights by region or food processing methods), we avoided this in favor of providing simple, standardized guidance for global use, as would presumably be needed for a metric for inclusion in global monitoring frameworks. In developing the GDQS, we ultimately attempted to balance the need for a valid metric responsive to diverse outcomes of nutrient adequacy and NCD risk in diverse settings, with that of an easily operationalized metric that can be in surveillance systems without adding undue burden to participants and surveyors that could hamper widespread uptake.

A third limitation of this study was the lack of cohort data in low-income countries. Although our analyses of large cohort studies in Mexico and the US provide strong evidence for validity of the GDQS against NCD outcomes, the growing burden of NCDs in low-income countries, and middle-income countries outside of the Americas, warrants further prospective analyses in these settings due to regional differences in the foods comprising each food group and differences in the prevailing health status of the population.

Our evaluation demonstrates that the GDQS meets an urgent need for a valid and robust metric capable of jointly capturing the immense and frequently coexisting burdens of nutrient inadequacy and diet-related NCD risk affecting many countries. We thus propose the GDQS and GDQS submetrics as appropriate metrics for integration in diverse surveillance and research platforms, including health and nutrition surveys, household surveys, and epidemiologic platforms. We envision numerous applications of the GDQS in practice, including standardized assessment and monitoring of population diet quality, comparing diet quality between populations and subpopulations, evaluating impacts of food-based nutrition programs, and designing and communicating policies and guidelines for improving diets. Global application of the GDQS as a unified and comparable diet quality measurement system would provide a strong evidence-base to support the coordinated approaches needed for reducing the global burden of both undernutrition and metabolic disease.

Research is warranted to translate and validate the GDQS for use in demographics beyond nonpregnant, nonlactating women of reproductive age; evaluate performance of the GDQS in longitudinal analysis of NCD outcomes in low-income countries; and validate primary assessment methods for capturing GDQS data in population surveys. Furthermore, whereas the scope of our research was limited to diet quality outcomes, food systems also play a critical role in planetary health. It would be valuable to explore relations between the GDQS and environmental impacts of food systems in different populations, to identify opportunities for developing novel metrics that jointly capture diet quality and food systems sustainability. Understanding these relationships may also inform dietary guidelines that optimize nutrition and planetary health (17).

## Supplementary Material

nxab244_Supplemental_Methods

## Notes

Funding for the research was provided by FHI Solutions, recipient of a Bill & Melinda Gates Foundation grant to support Intake – Center for Dietary Assessment. Support was also provided by grants from the Bill and Melinda Gates Foundation (OPP1179606), National Institutes of Health (U01 CA176726), and Wellcome Trust (083707, 084774, 084754, and 070797).

Author disclosures: TTF is an associate editor of the *Journal of Nutrition* and played no role in the Journal's evaluation of the manuscript. All other authors report no conflicts of interest.

Published in a supplement to the *Journal of Nutrition*. Publication costs for this supplement were funded by the Bill & Melinda Gates Foundation in conjunction with FHI Solutions, recipient of a Bill & Melinda Gates Foundation grant to support Intake – Center for Dietary Assessment. The opinions expressed in this publication are those of the authors and are not attributable to the sponsors or the publisher, Editor, or Editorial Board of the Journal of Nutrition. The Supplement Coordinators for the supplement publication were Megan Deitchler, Intake – Center for Dietary Assessment at FHI Solutions, Washington, DC; and Sabri Bromage, Harvard T.H. Chan School of Public Health, Boston, MA.

Supplemental Methods are available from the “Supplementary data” link in the online posting of the article and from the same link in the online table of contents at https://academic.oup.com/jn/.

The GDQS research initiative was launched by Intake – Center for Dietary Assessment. The research was led by Harvard T.H. Chan School of Public Health, Department of Nutrition and carried out in collaboration with researchers at the National Public Health Institute (INSP), Mexico. Funding for the research was provided by FHI Solutions, recipient of a Bill & Melinda Gates Foundation grant to support Intake – Center for Dietary Assessment.

Abbreviations used: AHEI-2010, Alternative Health Eating Index – 2010; ATP, Adult Treatment Panel; GDQS, Global Diet Quality Score; GDQS-, GDQS Negative Submetric; GDQS+, GDQS Positive Submetric; LMIC, low- and middle- income countries; MDD-W, Minimum Dietary Diversity - Women; MetS, metabolic syndrome; MUAC, mid–upper arm circumference; NCD, noncommunicable disease; NHS II, Nurses’ Health Study II; PDQS, Prime Diet Quality Score; WRA, women of reproductive age; 24HR, 24-h recall.
