# Supplementary material for: Development and Validation of a Novel Food-Based Global Diet Quality Score (GDQS)
Source: J Nutr. 2021 Oct 23;151(Suppl 2):75S–92S. doi: 10.1093/jn/nxab244 (PMC8542096; doi:10.1093/jn/nxab244)
Supplement: nxab244_Supplemental_Methods [file nxab244_supplemental_methods.docx]

**Online Supplementary Material** for “Development and validation of a novel food-based Global Diet Quality Score” (authors: Bromage S, Batis C, Bhupathiraju SN, Fawzi WW, Fung TT, Li Y, Deitchler M, Angulo E, Birk N, Castellanos-Gutiérrez A, He Y, Fang Y, Matsuzaki M, Zhang Y_,_ Moursi M, Gicevic S, Holmes MD, Isanaka S, Kinra S, Sachs SE, Stampfer MJ, Stern D, Willett WC)

**Supplemental Methods:** Scientific basis and operational definition of GDQS food groups

*Citrus fruits, cruciferous vegetables, dark green leafy vegetables, deep orange fruits, deep orange tubers, deep orange vegetables, legumes, other fruits, other vegetables (healthy scoring)*

Fruits and vegetables are excellent sources of vitamins, minerals, dietary fiber, and phytochemicals such as polyphenols, and consumption of fruits and vegetables is associated with lower blood pressure, lower blood sugar, weight loss, and reduced incidence of cardiovascular disease, certain cancers, and other diseases [1-4]. In defining fruits and vegetables, we included only whole fruits and vegetables, not juices or spreads (e.g. jam and marmalade) which have different nutritional properties. We also excluded coconuts and their products, such as coconut milk and oil, given their high saturated fat content. In some cases, we classified actual fruits as vegetables (e.g., tomato) and actual vegetables as fruits (e.g., rhubarb) if that made more sense based on nutritional (and to some extent, culinary) characteristics.

Dark green leafy vegetables, cruciferous vegetables, and citrus fruits in particular appear to make important contributions to decreased risk of heart attack and stroke based on analysis of large cohort studies [5]; for these reasons, and because these food groups represent distinct families of fruits and vegetables that are convenient to distinguish in data collection, we deliberately separated these groups in the food list. To define citrus fruits and cruciferous vegetables, we used the genus *Citrus* and family *Brassicaceae*, respectively; while citrus fruits are particularly high in vitamin C, we did not apply a particular vitamin C cutoff to define this group as citrus fruits also contain other important components, and because other types of fruits and vegetables also contain significant amounts of vitamin C. We defined dark green leafy vegetables using a cutoff of ≥120 retinol equivalents per 100g [6].

Deep orange fruits, deep orange vegetables, and deep orange tubers are scored as healthy food groups owing to their high content of carotenoids and other vitamins and minerals. We created these three groups by dividing the Prime Diet Quality Score (PDQS) food group of “deep orange fruits and vegetables”. These foods are distinguished from one another in the GDQS given their different nutritional properties and, in populations consuming large amounts of fruits and vegetables, it would be helpful to have a larger number of categories to include them in to allow their distinct contributions to diet quality to be accounted for. We also separated these categories to allow users of the metric to derive total fruits and, independently, total vegetables, which could be indicators of interest (for these reasons, we also retained the PDQS groups of “other fruits” and “other vegetables”). We defined deep orange foods using the same ≥120 retinol equivalents per 100g cutoff used for dark green leafy vegetables [6]. Deep orange tubers include variants biofortified with vitamin A.

Legumes were separated from other vegetables on the basis of their particularly high content of important macronutrients and micronutrients, including plant protein; complex carbohydrates; soluble and insoluble fiber; folate; iron; phosphorus; and polyunsaturated fatty acids, including linoleic and oleic acids. Consumption of legumes also appears to be preventive against various chronic diseases [7-8]. In defining legumes, we included products arising from legumes, such as tofu and soymilk, but did not include bean sprouts (which we classified in other vegetables) or groundnuts, such as peanuts and bambara nuts, which are technically legumes but which have nutritional properties more similar to nuts (and are thus grouped with nuts and seeds).

*Nuts and seeds (healthy scoring)*

Nuts and seeds are included on the basis of their protein, vitamin, mineral, and omega-3 fatty acid profiles, and findings from large cohort studies showing that nut consumption is associated with improved lipid profile and lower risk of heart attack, heart disease mortality, type 2 diabetes, blood clots, and erratic heart rhythm [9-10]. Seeds in this category are distinguished from those that grow in pods (which belong in the legumes category) based on their nutritional properties, which are more similar to those of nuts. For the same reason, groundnuts are included in this category rather than in legumes. The nuts and seeds group also includes nut-based butters and other products (except oils), and seeds that are used as spices (only when used in their whole form, not their powdered form).

*White roots and tubers (unhealthy scoring)*

While important for energy adequacy in LMICs (particularly in Sub-Saharan Africa), we classified white roots and tubers as unhealthy given their relatively low micronutrient content, comparatively high glycemic index and effects on blood sugar (especially as compared with other staple groups including deep orange tubers, whole grains, and legumes), and evidence from large cohort studies showing that increased consumption of potatoes contributes to weight gain and increased diabetes risk while decreased consumption is associated with opposite effects [11-12]. We defined white roots and tubers as those containing less than 120 retinol equivalents per 100g [6] (note that these foods may actually be beige or otherwise not strictly white in color). This group includes flours made from these foods, such as potato or cassava flour.

*Liquid oils (healthy scoring)*

Oils that are liquid at room temperature are included as a healthy-scored food group given their high contents of poly- and monounsaturated fatty acids, which in some cases include significant amounts of omega-3 alpha-linolenic acid. Synthesis of experimental evidence has shown that replacement of carbohydrates with polyunsaturated and monounsaturated fats decreases concentrations of LDL cholesterol and increases HDL cholesterol [13], lowers blood pressure, and reduces cardiovascular risk [14]. In defining liquid oils, we included palm olein, liquid palm kernel oil, and liquid coconut oil on the basis of their relatively unsaturated lipid profile in comparison with solid or semisolid variants of these products, and to simplify data collection. This category does not include liquid oil used to deep fry foods that are purchased, but does include liquid oil used to deep fry foods that are prepared at home.

*Poultry and game meat (healthy scoring)*

Poultry typically contains smaller amounts of saturated fat compared with red meat, and is a rich source of animal protein. Epidemiologic evidence indicates that consumption of poultry is associated with decreased risk of cardiovascular risk factors and mortality [15-17]. This category refers to unprocessed poultry and game only, including unprocessed poultry organs, while processed poultry and processed poultry organs belong in the processed meat group. (We defined processing using the International Agency for Research on Cancer [IARC] definition described in the “Red and processed meat” section below.) Considering their importance for nutrition in LMICs in particular [], we also included a large variety of game meats in this category, which are also rich sources of lean animal protein. Game meat includes different kinds of wild animals and bush meat, such as primates, rodents, canines, felines, marsupials, leporids (rabbits and hares), wild boar, bats, bears, semiaquatic mammals (including otters and beavers), undomesticated ungulates, reptiles (aquatic and terrestrial), and amphibians.

*Eggs (healthy scoring)*

Although eggs contain high amounts of cholesterol, dietary cholesterol is only weakly correlated with that in the blood, and most of the fatty acids contained in eggs are healthy monounsaturated or polyunsaturated ones. Consumption of eggs is not associated with increased risk of heart disease in healthy individuals [19], and while high egg intake may modestly increase risk of increase diabetes or cardiovascular risk [20], particularly in those with existing NCDs or NCD risk factors [21], eggs are also rich sources of vitamins, minerals, and animal protein, which is especially important to consider in the LMIC context. For these reasons, on balance, we considered eggs to be a healthy dietary food group. Eggs consumed from any animal regardless of the manner of preparation of the egg (e.g., boiled, fried, etc.) are included in this category. This category does not include mayonnaise.

*Fish and shellfish (healthy scoring)*

Fish are an excellent source of animal protein and selenium, and a major source of omega-3 fatty acids and dietary vitamin D in diets around the world. Fish consumption is associated with a reduced risk of heart disease mortality and healthy prenatal and infant neurological development, and may also reduce the risk of stroke, depression, Alzheimer’s disease, and other chronic conditions [22-23]. While fish can contain toxins such as mercury and polychlorinated biphenyls, evidence regarding the association between fish consumption and health conditions resulting from toxin exposure is controversial, and such exposure is unlikely to significantly outweigh the benefits of eating fish for the majority of persons and consumption levels.

In defining the fish group, we included all kinds of fish based on their phylogenetic classification (including sharks, eels, and rays) and on the observation that all of these are rich in omega-3 fatty acids. We also included other non-fish seafood that contain significant concentrations of omega-3 fatty acids, including shellfish (aquatic mollusks and crustaceans, including sea snails), jellyfish, cetaceans (whales and dolphins), pinnipeds (seals and walruses), but not echinoderms (sea urchins and sea cucumbers). We further included fish organs in this group, and, unlike in the red meat and poultry categories, we included both processed and unprocessed fish.

*Red meat (scored as unhealthy in excessive amounts) and processed meat (unhealthy scoring)*

Regular consumption of red meat – especially processed red meat – is linked to an increased risk of heart disease, stroke and death from cardiovascular disease or other causes, and replacing red and processed meat with healthy protein sources appears to reduce these risks [15-17]. Consumption of red meat (particularly processed meat) is also associated with a higher risk of type 2 diabetes and certain cancers [24-25]. However, red meat is also an excellent source of complete protein (protein made up of all 20 amino acids that the body requires) and some vitamins and minerals. Therefore, although we give unhealthy scoring to processed meat, red meat is scored as unhealthy only in excessive amounts to recognize the important potential contribution of modest meat consumption to nutrient intakes in LMICS while also recognizing the risks associated with higher levels of consumption that may occur in LMICs as well as in high-income countries.

We defined red meat as that belonging to domesticated animals (i.e., not game meat) and according to nutritional (versus culinary) characteristics, and therefore included in this group certain meats (such as pork and lamb) that are sometimes otherwise classified as white meat but whose fatty acid profile is more similar to beef, horse, and mutton, and less similar to poultry and game. As with poultry, we included organs (including blood) in the definition of red meat. We defined processed meat to include processed red meat, processed poultry, and processed game meat (but not processed fish, which is retained in the fish group). We defined processing according to the IARC definition: “meat that has been transformed through salting, curing, fermentation, smoking or other processes to enhance flavor or improve preservation.” This is a broad definition, but we adopted it given limited evidence that non-industrially or minimally-processed meats are on the whole significantly healthier than more highly processed ones. In data collection, probes may be necessary to determine whether a meat would be classified as processed based on this definition.

*Low fat dairy (healthy scoring) and high fat dairy (scored as unhealthy in excessive amounts)*

Milk and dairy products are rich sources of calcium and other micronutrients, as well as protein [26]. However, milk and dairy can also be high in saturated fat, which is a risk factor for cardiovascular disease through its effects on blood lipids [13]. Furthermore, analysis of three large cohorts with more than 30 years of follow-up found whole milk consumption to be associated with higher total mortality, while consumption of low fat milk and cheese was not [27] We therefore scored low fat dairy as a healthy group and high fat dairy as unhealthy in excessive amounts, to recognize the latter’s potential contribution to nutrient intakes (especially in LMICs) at modest levels of consumption, while also recognizing its potential contribution to NCD risk associated with higher levels of consumption.

We used ≤2% milk fat as the defining cutoff between low fat and high fat milk and dairy products. Low fat dairy includes reduced fat products, as well as those naturally low in fat. High-fat dairy does not include butter or clarified butter, which we considered to belong with solid fats (which are not scored) given these foods’ comparatively low protein content. High fat dairy also does not include ice cream or whipped cream (which belong in sweets and ice cream). Chocolate milk and other flavored milks are classified as dairy products and not with sugar-sweetened beverages; however, sugar added to these drinks should, when possible, be counted toward the sweets and ice cream group. Conversely, tea and coffee to which milk has been added should not be counted as dairy, but the milk added to them should be counted as such (if ingredient information is available to do so).

*Purchased deep fried foods (unhealthy scoring)*

Analyses of large U.S. cohorts has found that fried foods eaten away from home are related to a higher risk of diabetes and other poor health outcomes [28]. One reason for this finding may be the types of fat used to fry foods at home versus those used away from home: foods fried away from home may be more often prepared using fats or oils that contain higher proportions of saturated or trans fatty acids due to factors related to cost or taste, or due to the potential for oils to be used and reused in restaurant and other food service settings (which may lead to the formation of trans isomers). A second reason is that fried foods away from home may more frequently be fast foods that are associated with other nutritional risks (including high amounts of added sodium and other potentially deleterious additives or cooking practices).

For these reasons, deep fried foods that are purchased are scored as unhealthy, while deep fried foods prepared at home are not scored. We specifically highlight deep fried foods (defined as those fried in an amount of fat or oil sufficient to cover the food completely) rather than simply “fried” foods to target foods that are subject to a greater intensity of frying and to reduce misclassification that would otherwise be more problematic in attempting to determine whether foods were shallow/pan fried. Similarly, we highlight deep fried foods that are purchased (rather than simply eaten or prepared away from home) to reduce misclassification that would otherwise be more problematic in attempting to determine where foods were eaten or prepared, and because homemade deep fried foods eaten away from home are typically less deleterious than purchased deep fried foods regardless of where they are consumed.

Note: To also capture the nutritional contributions of the “underlying” foods that are deep fried, we “double-count” deep fried foods that are purchased by additionally scoring them according to other food group(s) that they belong to. Additionally: If a food that is deep fried and purchased is fried using liquid oil, the oil is not counted toward the liquid oils food group (by virtue of these oils’ comparatively deleterious fatty acid profile), while if the food is deep fried at home using liquid oil, the oil does count toward the liquid oil group. In data collection, probes may be necessary to determine whether a fried food was homemade or purchased, or deep fried or shallow/pan fried; when such probes are not possible, fried foods that are typically purchased or deep fried in a given context may be classified as such.

*Box: Approach for classifying foods deep fried at home or purchased*

| **Example: Potatoes deep fried at home**   - Food mass does not count toward deep fried foods group. - Food mass does count toward white roots and tubers group. - If deep fried in liquid oil, liquid oil mass does count toward liquid oil group. | **Example: Purchased deep fried French fries**   - Food mass does count toward deep fried foods group. - Food mass does count toward white roots and tubers group. - If fried in liquid oil, liquid oil mass does not count toward liquid oil group. |
| --- | --- |

*Juice, sugar-sweetened beverages, and sweets and ice cream (unhealthy scoring)*

Juice may contain significant concentrations of nutrients, including vitamins and fiber, if most of the whole fruit and/or vegetable is blended into the beverage (although whole fruits are still more conducive to longer-term satiety). Juices that have had most of the pulp and flesh removed are comparatively lacking in nutrients and more likely to cause a spike in blood glucose due to their low fiber content, even in the absence of added sugar, and this further leads to decreased satiety. Consequentially, consumption of fruit juice is associated with weight gain and an increased risk of type 2 diabetes [12,29], and juice is scored as an unhealthy group. In defining this group, we included any unsweetened or sweetened drinks that are at least part or wholly composed of fruit juice; we also included smoothies made of whole fruit (although these retain the fruit’s fiber and nutrient content, they do not provide the same degree of long-term satiety as whole fruit).

Sweetened drinks that do not contain any juice are classified as sugar-sweetened beverages. They include sodas, energy drinks, and sports drinks, and are also scored as unhealthy. These beverages are associated with weight gain and an increased risk of type 2 diabetes, heart disease, poor bone health, and premature death [30-32]. Beverages made using low-calorie sweeteners, such as diet sodas, are also included in this group. Sweetened tea and coffee and dairy or cereal-based drinks are not included in this group; despite larger amounts of sugar often added to tea and coffee in some parts of the world than others, these drinks generally do not contain the large bolus dose of sugar that is provided by sodas, and tea and coffee may also provide modest health benefits. If ingredient information is available, the sugar added to these drinks should be counted toward the separate category of sweets and ice cream.

Sugar-sweetened foods that are not beverages, including sugar and other caloric sweeteners, sweets, and ice cream, are also scored as unhealthy in a separate category for sweets and ice cream. This category includes whipped cream.

*Whole grains (healthy scoring) and refined grains and baked goods (unhealthy scoring)*

Whole grains are rich sources of fiber, B vitamins, minerals, antioxidants, phytochemicals, and plant protein. Concentrations of these components are comparatively reduced in refined grains because of the refining process, and this is reflected in these food groups’ different effects on health. Whole grain consumption is associated with a reduced risk of death from cardiovascular disease, cancer, and diverse inflammatory and infectious causes, and consumption of whole grains in place of refined grains is associated with substantially improved lipid and insulin profile [33-36]. Based on these findings, we scored whole grains and whole grain products as healthy food group and refined grains and their products as unhealthy food groups.

Grain products can be distinguished as whole vs. refined using information on local flour processing methods. In the data collection process, because respondents may often be unaware of whether grain products they consumed were whole or refined, asking about the color of the food (“brown” or “white”) is a convenient way to operationalize the metric. Grain products with significant amounts of added sugar (qualitatively defined as amounts that typically result in a detectably sweet taste) are classified in the sweets group, and not as whole or refined grains. In cases where data on the amount of sugar can be disaggregated from the amount of grain (such as in sweetened cereal-based beverages), the grain should be counted toward the whole or refined grain group, as appropriate, and the sugar counted toward the sweets group.

We recognize that modern maize, which is widely consumed in Latin American countries, has been bred for yield, resulting in high ratio of starch to fiber and micronutrients. Regions with high maize consumption should thus interpret the classification of whole grain maize products with caution, while awaiting further data on consumption of maize in relation to health outcomes in these populations. In similar instances (or instances in which the processing method of a grain is unknown), it may be appropriate to classify grains or grain products based on their nutrient or fiber density (for example, classifying grain products as whole when the fiber to carbohydrate ratio is ≥0.1 [37]).

*Semisolid and solid fats (not scored)*

Although hydrogenated oils are potentially significant sources of trans fat (intake of which is a strong predictor of heart disease and that contributes to insulin resistance, inflammation, and worsened lipid profile), the concentrations of trans fatty acids in hydrogenated oils varies widely across the world due to differences in food production practices and regulations. We therefore reasoned that including these foods in the GDQS may therefore lead to inconsistent validity against diet quality outcomes between populations and reduce comparability of results. Furthermore, while semisolid and solid fats in general contain high amounts of saturated fat, which is an important dietary component to consider in relation to NCD risk, reduction in saturated fat is itself not a strong predictor of decreased mortality risk unless it is replaced by healthier fats [38-40] (we do give points for liquid plant oils, so those who replace solid fats with healthy oils in their diets will receive credit).

*Insects (not scored)*

Insects may be important contributors to nutrient intakes and health in some populations, and may become even more important in the future [41-42]. However, due to a lack of information on health effects of consuming insects, and insects’ potentially dissimilar nutritional and culinary properties to other food groups, and, we did not include insects as its own or as part of another food group.

*Other foods: fortified foods, alcoholic beverages, coffee, tea (not scored)*

As a simple metric of diet quality, the GDQS does not intend to capture information related to the consumption of nutrient fortificants or alcoholic beverages, despite their role in health. Fortified foods should be classified in the food group that corresponds to the unfortified version of that food (e.g., orange juice fortified with calcium should be classified in the juice category; liquid oil fortified with vitamin A should be classified in the liquid oil category, etc.). Coffee and tea are also excluded from scoring; while these generally appear to provide modest health benefits, conclusions as to the overall importance of their contribution remain mixed.

**References**

1. Appel LJ, Moore TJ, Obarzanek E, Vollmer WM, Svetkey LP, Sacks FM, Bray GA, Vogt TM, Cutler JA, Windhauser MM, Lin PH. A clinical trial of the effects of dietary patterns on blood pressure. New England Journal of Medicine. 1997 Apr 17;336(16):1117-24.
2. Wiseman M. The Second World Cancer Research Fund/American Institute for Cancer Research Expert Report. Food, Nutrition, Physical Activity, and the Prevention of Cancer: A Global Perspective: Nutrition Society and BAPEN Medical Symposium on ‘Nutrition support in cancer therapy’. Proceedings of the Nutrition Society. 2008 Aug;67(3):253-6.
3. Wang X, Ouyang Y, Liu J, Zhu M, Zhao G, Bao W, Hu FB. Fruit and vegetable consumption and mortality from all causes, cardiovascular disease, and cancer: systematic review and dose-response meta-analysis of prospective cohort studies. [BMJ](https://www.bmj.com/content/349/bmj.g4490.full.pdf+html). 2014 Jul 29;349:g4490.
4. Bertoia ML, Mukamal KJ, Cahill LE, Hou T, Ludwig DS, Mozaffarian D, Willett WC, Hu FB, Rimm EB. Changes in intake of fruits and vegetables and weight change in United States men and women followed for up to 24 years: analysis from three prospective cohort studies. PloS medicine. 2015 Sep 22;12(9):e1001878.
5. Hung HC, Joshipura KJ, Jiang R, Hu FB, Hunter D, Smith-Warner SA, Colditz GA, Rosner B, Spiegelman D, Willett WC. Fruit and vegetable intake and risk of major chronic disease. Journal of the National Cancer Institute. 2004 Nov 3;96(21):1577-84.
6. FAO and FHI 360. 2016. Minimum Dietary Diversity for Women: A Guide for Measurement. Rome: FAO
7. Afshin A, Micha R, Khatibzadeh S, Mozaffarian D. Consumption of nuts and legumes and risk of incident ischemic heart disease, stroke, and diabetes: a systematic review and meta-analysis. The American journal of clinical nutrition. 2014 Jun 4;100(1):278-88.
8. Rebello CJ, Greenway FL, Finley JW. A review of the nutritional value of legumes and their effects on obesity and its related co‐morbidities. Obesity Reviews. 2014 May;15(5):392-407.
9. Guasch-Ferré M, Liu X, Malik VS, Sun Q, Willett WC, Manson JE, Rexrode KM, Li Y, Hu FB, Bhupathiraju SN. Nut consumption and risk of cardiovascular disease. Journal of the American College of Cardiology. 2017 Nov 21;70(20):2519-32.
10. Liu G, Guasch-Ferré M, Hu Y, Li Y, Hu FB, Rimm EB, Manson JE, Rexrode KM, Sun Q. Nut Consumption in Relation to Cardiovascular Disease Incidence and Mortality Among Patients With Diabetes Mellitus. Circ Res. 2019 Mar 15;124(6):920-929.
11. Barclay AW, Petocz P, McMillan-Price J, Flood VM, Prvan T, Mitchell P, Brand-Miller JC. Glycemic index, glycemic load, and chronic disease risk--a meta-analysis of observational studies. Am J Clin Nutr. 2008 Mar;87(3):627-37.
12. Mozaffarian D, Hao T, Rimm EB, Willett WC, Hu FB. Changes in diet and lifestyle and long-term weight gain in women and men. N Engl J Med. 2011 Jun 23;364(25):2392-404.
13. Mensink RP, Zock PL, Kester AD, Katan MB. Effects of dietary fatty acids and carbohydrates on the ratio of serum total to HDL cholesterol and on serum lipids and apolipoproteins: a meta-analysis of 60 controlled trials. Am J Clin Nutr. 2003 May;77(5):1146-55.
14. Appel LJ, Sacks FM, Carey VJ, Obarzanek E, Swain JF, Miller ER 3rd, Conlin PR, Erlinger TP, Rosner BA, Laranjo NM, Charleston J, McCarron P, Bishop LM; OmniHeart Collaborative Research Group. Effects of protein, monounsaturated fat, and carbohydrate intake on blood pressure and serum lipids: results of the OmniHeart randomized trial. JAMA. 2005 Nov 16;294(19):2455-64.
15. Bernstein AM, Sun Q, Hu FB, Stampfer MJ, Manson JE, Willett WC. Major dietary protein sources and risk of coronary heart disease in women. Circulation. 2010 Aug 31;122(9):876-83.
16. Bernstein AM, Pan A, Rexrode KM, Stampfer M, Hu FB, Mozaffarian D, Willett WC. Dietary protein sources and the risk of stroke in men and women. Stroke. 2011 Jan 1:STROKEAHA-111.
17. Pan A, Sun Q, Bernstein AM, Schulze MB, Manson JE, Stampfer MJ, Willett WC, Hu FB. Red meat consumption and mortality: results from 2 prospective cohort studies. Archives of internal medicine. 2012 Apr 9;172(7):555-63.
18. Ripple WJ, Abernethy K, Betts MG, Chapron G, Dirzo R, Galetti M, Levi T, Lindsey PA, Macdonald DW, Machovina B, Newsome TM, Peres CA, Wallach AD, Wolf C, Young H. Bushmeat hunting and extinction risk to the world’s mammals. R Soc Open Sci. 2016 Oct 19;3(10):160498.
19. Hu FB, Stampfer MJ, Rimm EB, Manson JE, Ascherio A, Colditz GA, Rosner BA, Spiegelman D, Speizer FE, Sacks FM, Hennekens CH, Willett WC. A prospective study of egg consumption and risk of cardiovascular disease in men and women. JAMA. 1999 Apr 21;281(15):1387-94.
20. Djoussé L, Gaziano JM. Egg consumption and risk of heart failure in the Physicians' Health Study. Circulation. 2008 Jan 29;117(4):512-6.
21. Shin JY, Xun P, Nakamura Y, He K. Egg consumption in relation to risk of cardiovascular disease and diabetes: a systematic review and meta-analysis. Am J Clin Nutr. 2013 Jul;98(1):146-59.
22. Mozaffarian D, Rimm EB. Fish intake, contaminants, and human health: evaluating the risks and the benefits. JAMA. 2006 Oct 18;296(15):1885-99.
23. Raji CA, Erickson KI, Lopez OL, Kuller LH, Gach HM, Thompson PM, Riverol M, Becker JT. Regular fish consumption and age-related brain gray matter loss. Am J Prev Med. 2014 Oct;47(4):444-51.
24. Pan A, Sun Q, Bernstein AM, Manson JE, Willett WC, Hu FB. Changes in red meat consumption and subsequent risk of type 2 diabetes mellitus: three cohorts of US men and women. JAMA internal medicine. 2013 Jul 22;173(14):1328-35.
25. Bouvard V, Loomis D, Guyton KZ, Grosse Y, El Ghissassi F, Benbrahim-Tallaa L, Guha N, Mattock H, Straif K. Carcinogenicity of consumption of red and processed meat. The Lancet Oncology. 2015 Dec 1;16(16):1599-600.
26. Willett WC, Ludwig DS. Milk and Health. N Engl J Med. 2020 Feb 13;382(7):644-654.
27. Ding M, Li J, Qi L, Ellervik C, Zhang X, Manson JE, Stampfer M, Chavarro JE, Rexrode KM, Kraft P, Chasman D, Willett WC, Hu FB. Associations of dairy intake with risk of mortality in women and men: three prospective cohort studies. BMJ 2019;367:l6204.
28. Cahill LE, Pan A, Chiuve SE, Sun Q, Willett WC, Hu FB, Rimm EB. Fried-food consumption and risk of type 2 diabetes and coronary artery disease: a prospective study in 2 cohorts of US women and men. Am J Clin Nutr. 2014 Aug;100(2):667-75.
29. Muraki I, Imamura F, Manson JE, Hu FB, Willett WC, van Dam RM, Sun Q. Fruit consumption and risk of type 2 diabetes: results from three prospective longitudinal cohort studies. BMJ. 2013 Aug 28;347:f5001.
30. Field AE, Sonneville KR, Falbe J, Flint A, Haines J, Rosner B, Camargo CA Jr. Association of sports drinks with weight gain among adolescents and young adults. Obesity (Silver Spring). 2014 Oct;22(10):2238-43
31. Al-Shaar L, Vercammen K, Lu C, Richardson S, Tamez M, Mattei J. Health Effects and Public Health Concerns of Energy Drink Consumption in the United States: A Mini-Review. Front Public Health. 2017 Aug 31;5:225.
32. Malik VS, Li Y, Pan A, De Koning L, Schernhammer E, Willett WC, Hu FB. Long-Term Consumption of Sugar-Sweetened and Artificially Sweetened Beverages and Risk of Mortality in US Adults. Circulation. 2019 Apr 30;139(18):2113-2125.
33. Jacobs DR Jr, Andersen LF, Blomhoff R. Whole-grain consumption is associated with a reduced risk of noncardiovascular, noncancer death attributed to inflammatory diseases in the Iowa Women's Health Study. Am J Clin Nutr. 2007 Jun;85(6):1606-14.
34. Mellen PB, Walsh TF, Herrington DM. Whole grain intake and cardiovascular disease: a meta-analysis. Nutr Metab Cardiovasc Dis. 2008 May;18(4):283-90.
35. Aune D, Norat T, Romundstad P, Vatten LJ. Whole grain and refined grain consumption and the risk of type 2 diabetes: a systematic review and dose-response meta-analysis of cohort studies. Eur J Epidemiol. 2013 Nov;28(11):845-58.
36. Zong G, Gao A, Hu FB, Sun Q. Whole Grain Intake and Mortality From All Causes, Cardiovascular Disease, and Cancer: A Meta-Analysis of Prospective Cohort Studies. Circulation. 2016 Jun 14;133(24):2370-80.
37. Mozaffarian RS, Lee RM, Kennedy MA, Ludwig DS, Mozaffarian D and Gortmaker SL. Identifying whole grain foods: a comparison of different approaches for selecting more healthful whole grain products. Public Health Nutr. 2013;16:2255–64.
38. Astrup A, Dyerberg J, Elwood P, Hermansen K, Hu FB, Jakobsen MU, Kok FJ, Krauss RM, Lecerf JM, LeGrand P, Nestel P, Risérus U, Sanders T, Sinclair A, Stender S, Tholstrup T, Willett WC. The role of reducing intakes of saturated fat in the prevention of cardiovascular disease: where does the evidence stand in 2010? Am J Clin Nutr. 2011 Apr;93(4):684-8.
39. Farvid MS, Ding M, Pan A, Sun Q, Chiuve SE, Steffen LM, Willett WC, Hu FB. Dietary linoleic acid and risk of coronary heart disease: a systematic review and meta-analysis of prospective cohort studies. Circulation. 2014 Oct 28;130(18):1568-78.
40. Brassard D, Tessier-Grenier M, Allaire J, Rajendiran E, She Y, Ramprasath V, Gigleux I, Talbot D, Levy E, Tremblay A, Jones PJ, Couture P, Lamarche B. Comparison of the impact of SFAs from cheese and butter on cardiometabolic risk factors: a randomized controlled trial. Am J Clin Nutr. 2017 Apr;105(4):800-809.
41. Van Huis A, Dicke M, van Loon JJ. Insects to feed the world. Journal of Insects as Food and Feed. 2015 Jan 1;1(1):3-5.
42. Wendin K, Birch K, Olsson V. Insects as food: a review of sustainability, nutrition and consumer attitudes. InFood and Society Proceedings 2019 (pp. 145-152).
